# Interleukin-22 regulates *B3GNT7* expression to induce fucosylation of glycoproteins in intestinal epithelial cells

**DOI:** 10.1016/j.jbc.2021.101463

**Published:** 2021-12-02

**Authors:** Daniela J. Carroll, Mary W.N. Burns, Lynda Mottram, Daniel C. Propheter, Andrew Boucher, Gabrielle M. Lessen, Ashwani Kumar, Stacy A. Malaker, Chao Xing, Lora V. Hooper, Ulf Yrlid, Jennifer J. Kohler

**Affiliations:** 1 Department of Biochemistry, University of Texas Southwestern Medical Center, Dallas, Texas, USA; 2 Department of Microbiology and Immunology, Institute of Biomedicine, University of Gothenburg, Gothenburg, Sweden; 3 Department of Immunology, The University of Texas Southwestern Medical Center, Dallas, Texas, USA; 4 Eugene McDermott Center for Human Growth and Development, The University of Texas Southwestern Medical Center, Dallas, Texas, USA; 5 Department of Chemistry, Yale University, New Haven, Connecticut, USA; 6 Department of Bioinformatics, The University of Texas Southwestern Medical Center, Dallas, Texas, USA; 7 Department of Population and Data Sciences, The University of Texas Southwestern Medical Center, Dallas, Texas, USA; 8 Howard Hughes Medical Institute, Department of Immunology, The University of Texas Southwestern Medical Center, Dallas, Texas, USA

**Keywords:** IL-22, B3GNT7, glycosylation, fucosylation, glycosyltransferase, intestinal epithelium, 2F-Fuc, 2-fluoro-peracetyl-fucose, B3GNT7, β1-3-*N*-acetylglucosaminyltransferase 7, FUT, fucosyltransferase, Gal, galactose, GalNAc, *N*-acetylgalactosamine, GDP, guanosine diphosphate, GlcNAc, *N*-acetylglucosamine, IL, interleukin, IL-22Rα1, IL-22 receptor subunit alpha 1, ILC3, type 3 innate lymphoid cell, Le^x^, Lewis X, LEL, *Lycopersicon esculentum* lectin, LTL, *Lotus tetragonolobus* lectin, polyLacNAc, poly-*N*-acetyllactosamine, rh, recombinant human, RT-qPCR, quantitative real-time PCR, Ser, serine, STAT3, signal transducer and activator of transcription 3, Thr, threonine, UDP, uridine diphosphate, UEA I, *Ulex europaeus* agglutinin I

## Abstract

Interleukin (IL)-22 is a cytokine that plays a critical role in intestinal epithelial homeostasis. Its downstream functions are mediated through interaction with the heterodimeric IL-22 receptor and subsequent activation of signal transducer and activator of transcription 3 (STAT3). IL-22 signaling can induce transcription of genes necessary for intestinal epithelial cell proliferation, tissue regeneration, tight junction fortification, and antimicrobial production. Recent studies have also implicated IL-22 signaling in the regulation of intestinal epithelial fucosylation in mice. However, whether IL-22 regulates intestinal fucosylation in human intestinal epithelial cells and the molecular mechanisms that govern this process are unknown. Here, in experiments performed in human cell lines and human-derived enteroids, we show that IL-22 signaling regulates expression of the B3GNT7 transcript, which encodes a β1-3-*N*-acetylglucosaminyltransferase that can participate in the synthesis of poly-*N*-acetyllactosamine (polyLacNAc) chains. Additionally, we find that IL-22 signaling regulates levels of the α1-3-fucosylated Lewis X (Le^x^) blood group antigen, and that this glycan epitope is primarily displayed on *O*-glycosylated intestinal epithelial glycoproteins. Moreover, we show that increased expression of B3GNT7 alone is sufficient to promote increased display of Le^x^-decorated carbohydrate glycan structures primarily on *O*-glycosylated intestinal epithelial glycoproteins. Together, these data identify B3GNT7 as an intermediary in IL-22-dependent induction of fucosylation of glycoproteins and uncover a novel role for B3GNT7 in intestinal glycosylation.

Glycosylation is a ubiquitous posttranslational modification that produces a diverse array of cellular glycans. Glycan assembly is complex, and the process is driven by transcriptional regulation of a portfolio of cellular “glycogenes,” the relative abundance of glycoprotein substrates, and availability of nucleotide sugar donor substrates. Glycans are synthesized in a sequential manner, where glycosyltransferases with distinct substrate specificities extend structures by transferring activated sugars to acceptor substrates in an α- or β-linkage, generating a glycan repertoire that is displayed on cell surfaces, secreted proteins, and within certain organelles. Glycans participate in the regulation of diverse biological processes, including proper protein folding and secretion, cellular adhesion and signaling, and immune cell trafficking (1, 2, 3).

In the intestine, mucosal glycans are typically found attached to membrane-bound or secreted mucin-like glycoproteins through *O*-glycosidic linkages (*O*-glycosylation). GalNAc-type *O*-glycosylation begins in the Golgi apparatus with the transfer of *N*-acetylgalactosamine (GalNAc) to the hydroxyl group of a serine (Ser) or threonine (Thr) residue. This core structure can be elaborated into linear or branched structures. A common elaboration is the addition of poly-*N*-acetyllactosamine (polyLacNAc). polyLacNAc is a repeating copolymer of galactose (Gal) and *N*-acetylglucosamine (GlcNAc) produced by the concerted action of galactosyltransferases and GlcNAc-transferases. These extended *O*-glycans can be further decorated with sialic acid, fucose, sulfate groups, or ABO- or Lewis-type histo-blood group antigens, generating a wide diversity of possible structures (4). For example, 6-sulfation of GlcNAc residues results in keratan sulfate II (KS II) structures (5). Notably, mucosal *O*-glycans decorated with fucose have been identified as key regulators of both health and disease in the gut (6, 7).

L-fucose is a monosaccharide found in multiple classes of cell surface glycans. Incorporation of fucose into glycans is catalyzed by fucosyltransferases (FUTs). Thirteen human FUTs have been identified. FUTs catalyze the transfer of fucose from the guanosine diphosphate (GDP)—fucose donor to acceptor substrates in an α1-2-, α1-3-, α1-4- or α1-6-linkage, or α-linked to a serine or threonine side chain. Among these FUTs, ten are known to be involved in terminal fucosylation of glycan structures by decorating them with α1-2- (FUT1 and FUT2) or α1-3/4-linked fucose (FUT3–7 and FUT9–11) (8, 9).

Fucosylation is abundant in the mammalian gut and α1-2-fucosylation—primarily produced by Fut2—has emerged as a key regulator of commensal bacterial colonization and maintenance of bacterial symbiosis (10). Recent studies have explored the molecular mechanisms that control this process and implicate the interleukin (IL)-10 family member, IL-22, as the primary regulator of intestinal epithelial fucosylation in mice (10, 11, 12). In the intestine, IL-22 is produced by type 3 innate lymphoid cells (ILC3s) (13). The downstream function of IL-22 is mediated through ligation of its heterodimeric receptor comprised of IL-22 receptor subunit alpha 1 (IL-22Rα1) and IL-10R2 (14, 15), which signals through activation of signal transducer and activator of transcription 3 (STAT3) (16). IL-22 signaling plays a critical role in maintenance of the intestinal epithelial barrier by inducing genes necessary for intestinal epithelial cell proliferation, tissue regeneration, tight junction fortification, and induction of intestinal epithelial fucosylation. Despite the growing body of evidence that demonstrates the involvement of IL-22 in mouse intestinal fucosylation, whether IL-22 promotes intestinal fucosylation in humans and the glycosyltransferases involved remain to be explored.

Herein, we report that IL-22 signaling in human intestinal epithelial cells modulates expression of the B3GNT7 transcript and induces α1-3-fucosylation of glycoproteins, including those displaying mucosal *O*-linked glycans. We also show that overexpression of B3GNT7 is sufficient to cause increased fucosylation of *O*-linked glycans, thus identifying an unexpected mechanism by which intestinal fucosylation can be regulated.

## Results

### IL-22 regulates glycosyltransferase gene expression

**Figure 1:**
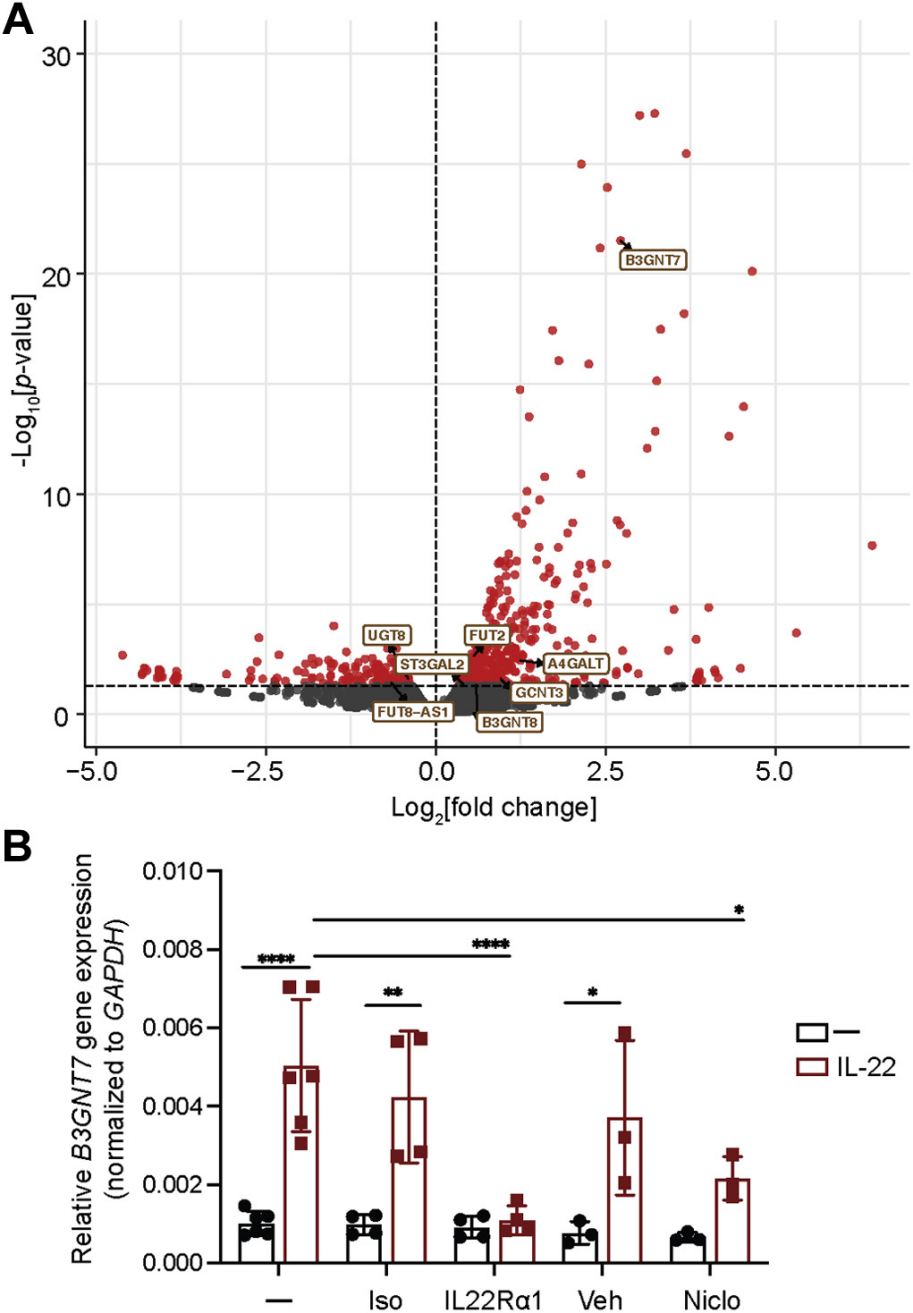
**IL-22 signaling regulates *B3GNT7* gene expression in human intestinal epithelial cells.***A*, differentiated Caco-2 BBe1 cells were treated with 10 ng/ml rhIL-22 for 4 h before subsequent RNA isolation for RNA-seq analysis. Volcano plot shows fold change (log_2_) in gene expression of differentiated Caco-2 BBe1 cells treated with rhIL-22 compared with untreated control, plotted against significance (−log_10_[*p* value]). Significant genes (*p* value < 0.05) are represented in red and significantly upregulated genes encoding glycosyltransferases are labeled in *brown*. Numerical values of significant genes are available in Table S1 (n = 3; *p* < 0.05). *B*, differentiated Caco-2 BBe1 cells were incubated with 5 μg/ml IL22Rα1 blocking antibody (IL22Rα1) or isotype control (Iso), or 0.5 μM niclosamide (Niclo) or DMSO (Veh) for 1 h before the addition of rhIL-22 (10 ng/ml) for 4 h before subsequent RNA isolation. *B3GNT7* gene expression was assessed using qRT-PCR, and data were normalized to *GAPDH* levels for each condition. *Symbols* represent individual biological replicates (n = 3–6), and error bars represent standard deviation. Statistical analysis was performed by one-way ANOVA with ∗ indicating *p* value between 0.01 and 0.05, ∗∗ indicating *p* value between 0.001 and 0.01, ∗∗∗ indicating *p* value between 0.001 and 0.0001, and ∗∗∗∗ indicating *p* value <0.0001. B3GNT7, β1-3-*N*-acetylglucosaminyltransferase 7; qRT-PCR, quantitative real-time PCR.

To interrogate the effect of IL-22 signaling on glycosyltransferase gene expression in human intestinal epithelial cells, we used differentiated Caco-2 BBe1 cells, a subclone of the Caco-2 human colorectal adenocarcinoma cell line that displays small-intestine-like morphology and biochemical properties (17, 18). Differentiated Caco-2 BBe1 cells were exposed to 10 ng/ml of recombinant human (rh) IL-22 for 4 h, and their global transcriptome was evaluated by RNA sequencing. In this model system, IL-22 significantly modulated the expression of 379 genes, including recognized IL-22-responsive transcripts (*TIFA*, *SOCS3*, *DUOX2*, *STAT3*, etc.) (Table S1). Consistent with mouse epithelial expression data (10, 11, 12), *in vitro* stimulation of human cells with IL-22 led to the induction of *FUT2.* Additionally, IL-22 induced significant changes in expression of eight additional genes encoding glycosyltransferases or their direct regulators (Fig. 1*A*). Among the glycosyltransferase genes regulated by IL-22, induction of *B3GNT7* showed the highest significance.

### IL-22 signaling regulates epithelial *B3GNT7* gene expression

To investigate the pathway by which IL-22 induces *B3GNT7* expression, we first corroborated the RNA-sequencing data by performing quantitative real-time PCR (RT-qPCR) analysis. We found that *B3GNT7* gene expression is upregulated by IL-22 in differentiated Caco-2 BBe1 cells (approximately fivefold increase; Fig. 1*B*). Increased *B3GNT7* expression was observed as soon as 2 h after IL-22 treatment (Fig. S1*A*), and no further increase in *B3GNT7* expression was achieved when the IL-22 concentration was increased to 100 ng/ml (Fig. S1*B*). To determine whether *B3GNT7* upregulation was dependent on activation of downstream IL-22 signaling, we first incubated differentiated Caco-2 BBe1 cells with an IL22Rα1 blocking antibody or isotype control. As shown in Figure 1*B*, IL-22-induced upregulation of *B3GNT7* gene expression was significantly inhibited (approximately 78% inhibition) in the presence of the IL22Rα1 blocking antibody, but not the isotype control. To evaluate the generality of these observations, we also examined T84 cells, which are derived from a lung metastasis of a colon carcinoma (19, 20). IL-22-dependent induction of *B3GNT7* expression was also observed in polarized T84 cells (appoximately 19-fold increase; Fig. S2). Polarized T84 cells were treated with an IL22Rα1 blocking antibody or isotype control, followed by IL-22 treatment. *B3GNT7* gene expression was increased by IL-22 treatment but significantly inhibited (approximately 67% inhibition) in the presence of the blocking antibody, but not the isotype control (Fig. S2).

Next, to determine whether *B3GNT7* gene expression was dependent on STAT3 activation, we incubated differentiated Caco-2 BBe1 cells with 0.5 μM niclosamide, a pharmacological inhibitor of STAT3 signaling (21), or vehicle control. We observed that the IL-22-dependent increase in *B3GNT7* gene expression was also inhibited in the presence of niclosamide (approximately 57% inhibition) (Fig. 1*B*). The moderate reduction in *B3GNT7* transcript expression was commensurate with the partial inhibition of STAT3 phosphorylation we observed for this concentration of niclosamide; unfortunately, higher niclosamide concentrations could not be examined due to toxicity.

**Figure 2:**
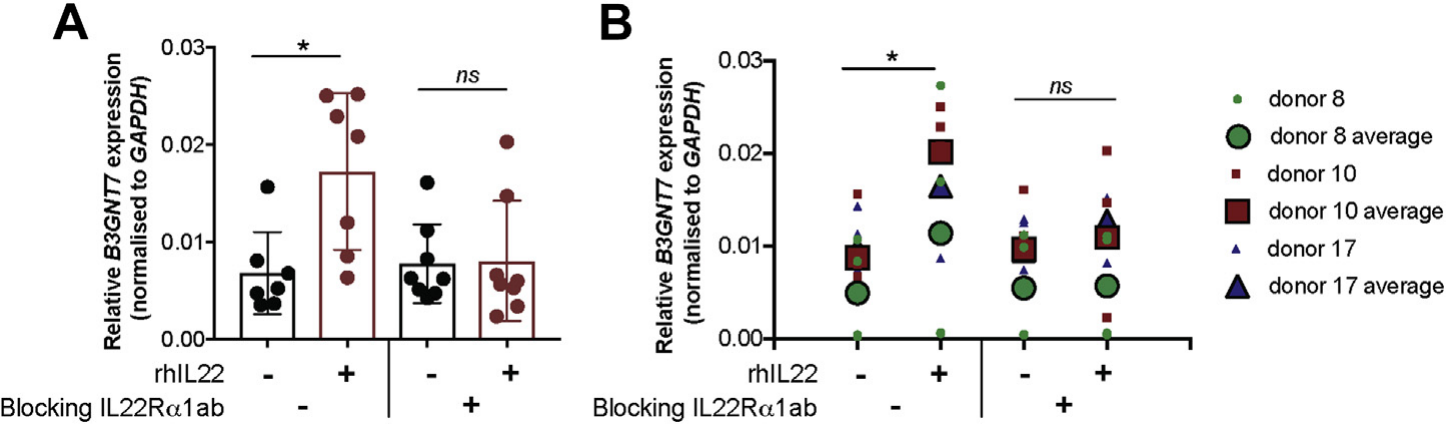
**IL-22 signaling regulates *B3GNT7* gene expression in human small intestinal enteroids.** Enteroid spheres were incubated with 5 μg/ml IL22Rα1 blocking antibody before the addition of 10 ng/ml rhIL-22 for 4 h. *B3GNT7* gene expression was quantified by qRT-PCR and normalized to *Gapdh* levels for each sample. *A*, analysis of *B3GNT7* gene expression from a single enteroid donor. *Symbols* represent biological replicates (n = 7–8). Bar graphs show the average, and error bars indicate standard deviation. *B*, analysis of *B3GNT7* gene expression in enteroids derived from three different donors. *Small symbols* represent biological replicates (n = 2–3 per donor); *large symbols* show the average for each donor line. Statistical significance was assessed by one-way ANOVA in panel *A* and nested one-way ANOVA in panel *B*. ∗*p* < 0.05. B3GNT7, β1-3-*N*-acetylglucosaminyltransferase 7; qRT-PCR,quantitative real-time PCR.

To investigate whether IL-22 signaling also regulates *B3GNT7* expression in nontransformed cells, we used human enteroids, which are derived from isolated small intestinal crypts. These enteroids can be passaged indefinitely while maintaining genetic and physiological features of the individual from which they were derived (22). We observed that IL-22 treatment resulted in increased *B3GNT7* expression in a cultured human enteroid line (approximately 2.5-fold increase; Fig. 2*A*). However, this increase was not observed when the IL22Rα1 blocking antibody was included. To test the generality of this observation, we repeated the experiment using enteroid lines derived from three different individuals, one of which was the line used in Figure 2*A*. While there was some variability among the lines, the combined data show a statistically significant increase in *B3GNT7* expression in response to IL-22 (Fig. 2*B*).

**Figure 3:**
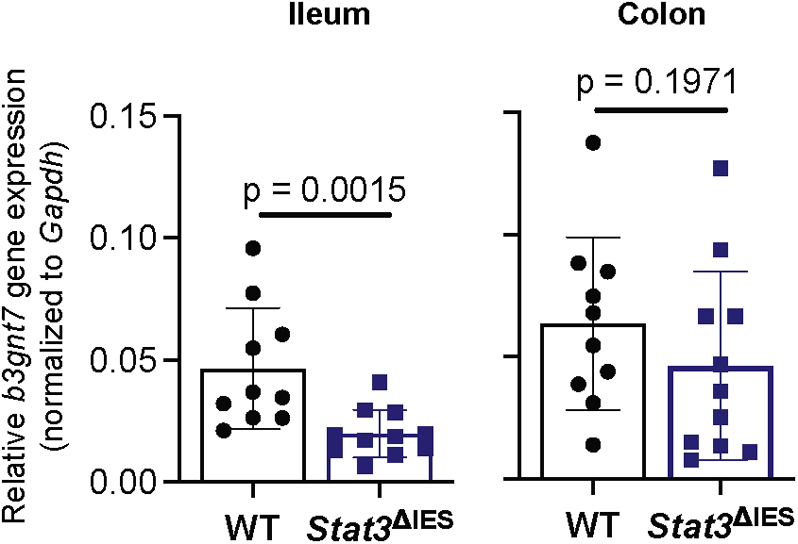
**Stat3 signaling regulates *b3gnt7* gene expression in mouse intestinal epithelium.***b3gnt7* gene expression in ileum and colon of WT and *Stat3*^*Δ*IEC^ mice was quantified by qRT-PCR and normalized to *Gapdh* levels for each sample. Data are from male and female mice, and *symbols* represent individual mice (n = 9 biologically independent animals for WT; n = 11 biologically independent animals for *Stat3*^*Δ*IEC^). Error bars show standard deviation. Statistical significance was assessed using a two-tailed Mann–Whitney test. B3GNT7, β1-3-*N*-acetylglucosaminyltransferase 7; qRT-PCR, quantitative real-time PCR.

Previously, it was observed that IL-22 regulates expression of *b3gnt7* in mouse colonic organoids, and this expression was IL22Rα1-dependent (12). Using intestinal epithelial tissue from mice lacking *Stat3* expression specifically in intestinal epithelial cells (*Stat3*^ΔIEC^) (23), we found that expression of *b3gnt7* was significantly impaired in the ileum, but not the colon, of these mice as compared with wild-type (WT) mice (Fig. 3).

Together, these results demonstrate that regulation of *B3GNT*7 gene expression appears to be conserved in humans and mice and *via* the IL-22-IL-22Rα1-STAT3 pathway. We also attempted to evaluate changes in B3GNT7 expression at the protein level but unfortunately were unable to detect endogenous B3GNT7 using any currently available antibodies.

### IL-22 induces epithelial α1-3-fucosylation

In mice, IL-22 initiates a rapid and substantial increase in intestinal epithelial fucosylation (10, 11, 12). However, whether IL-22 also promotes fucosylation in human intestinal epithelial cells has not yet been explored. Therefore, to assess the effect of IL-22 on overall fucosylation, we used fucose-recognizing lectins in lectin blots to probe the fucosylation status of glycoproteins from cell lysates. As shown in Fig. S3*A*, incubation of differentiated Caco-2 BBe1 cells with rhIL-22 for 48 h led to enhanced binding of *Lotus tetragonolobus lectin* (LTL), a lectin that specifically recognizes α1-3-linked fucose, but had no detectable effect on binding of *Ulex europaeus agglutinin I* (UEA I), a lectin that specifically recognizes α1-2-linked fucose. These findings were unexpected as previously published data showed that IL-22 enhances *Fut2* gene expression and subsequently induces small intestinal α1-2-fucosylation in mice in an IL22Rα1-dependent manner (11). Thus, we next validated our *in vitro* findings using epithelial tissue from *Stat3*^ΔIEC^ mice. First, consistent with published data, we found that *Fut2* gene expression was significantly reduced in the ileum of *Stat3*^ΔIEC^ mice (Fig. S3*B*), suggesting that *Fut2* gene expression in mice is also regulated *via* the IL-22-IL-22Rα1-STAT3 pathway. We also compared fucosylation patterns in WT *versus Stat3*^ΔIEC^ mice using UEA I and LTL to probe ileal tissue sections. As shown in Fig. S3*C*, both α1-2-fucosylation (examined using UEA I binding) and α1-3-fucosylation (examined using LTL binding) are present in the mouse small intestine, and *Stat3* deletion resulted in reduced binding of both lectins. Inclusion of 100 mM l-fucose in the binding buffer eliminated lectin staining, demonstrating the specificity of binding (Fig. S3*D*). Together, these results corroborate previous findings and demonstrate for the first time the ability for IL-22 signaling to regulate α1-3-fucosylation in both mouse and human intestinal epithelial cells.

**Figure 4:**
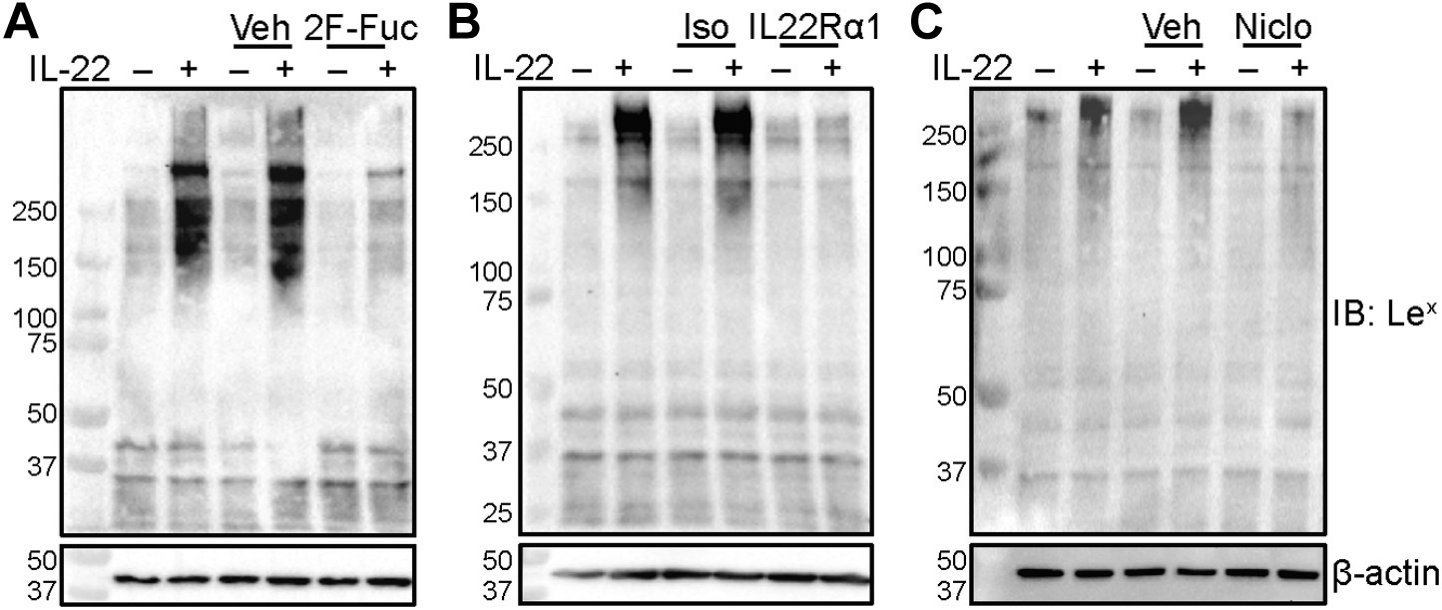
**IL-22 signaling regulates epithelial Le**^**x**^**antigen expression.** Differentiated Caco-2 BBe1 cells were incubated with (*A*) 200 μM 2F-Fuc or DMSO (Veh), (*B*) 5 μg/ml IL22Rα1 blocking antibody (IL22Rα1) or isotype control (Iso), or (*C*) 5 μM Niclosamide (Niclo) or DMSO (Veh) for 1 h before the addition of 10 ng/ml of rhIL-22 for 48 h before subsequent protein collection. Le^x^ antigen expression was assessed using Western blotting. Data presented are representative of at least three biological replicates.

Although LTL exhibits specificity for α1-3-fucose, it does not distinguish among multiple complex glycan epitopes that include α1-3-fucose (24). Lewis antigens are among the most commonly expressed α1-3-fucosylated carbohydrate epitopes on cell surfaces (25). Therefore, we next examined the effects of IL-22 on Lewis antigen expression in lysates from differentiated Caco-2 BBe1 cells. As shown in Figure 4*A*, exposure of differentiated Caco-2 BBe1 cells to rhIL-22 led to enhanced expression of the Lewis X (Le^x^) antigen. The increased Le^x^ antibody binding was inhibited in the presence of 2-fluoro-peracetyl-fucose (2F-Fuc), a metabolic inhibitor of fucosylation (26), demonstrating that increased antibody binding depends on fucosylated structures. To determine whether Le^x^ antigen expression was also dependent on IL-22 signaling, we also cultured differentiated Caco-2 BBe1 in the presence or absence of the IL22Rα1 blocking antibody or niclosamide. We observed that the IL-22-dependent increase in Le^x^ antigen expression was impaired following inhibition of IL-22 signaling (Fig. 4, *B* and *C*), suggesting that Le^x^ antigen expression, like *B3GNT7* expression, was also regulated *via* the IL-22-IL22Rα1-STAT3 pathway.

To assess the effects of IL-22 on the fucosylation of nontransformed cells, we performed a limited analysis of human-derived enteroids. Enteroids derived from three individuals were treated with rhIL-22, and changes in cell surface fucosylation were examined by flow cytometry (Fig. S4). IL-22 treatment of enteroids from all three individuals resulted in increased binding of the *Aleuria aurantia* lectin (AAL), which recognizes fucose in multiple linkages (27), and of the Le^x^-recognizing antibody. IL-22 did not result in increased UEA I binding to the enteroids. While the results were similar to those observed in differentiated Caco-2 BBe1 cells, sample availability was limited, and statistical analysis could not be performed. Additionally, variations in glycosylation among enteroid lines pose challenges in their analysis; therefore, subsequent experiments were performed solely in differentiated Caco-2 BBe1 cells.

### IL-22 induces epithelial fucosylation of *O*-linked glycans

**Figure 5:**
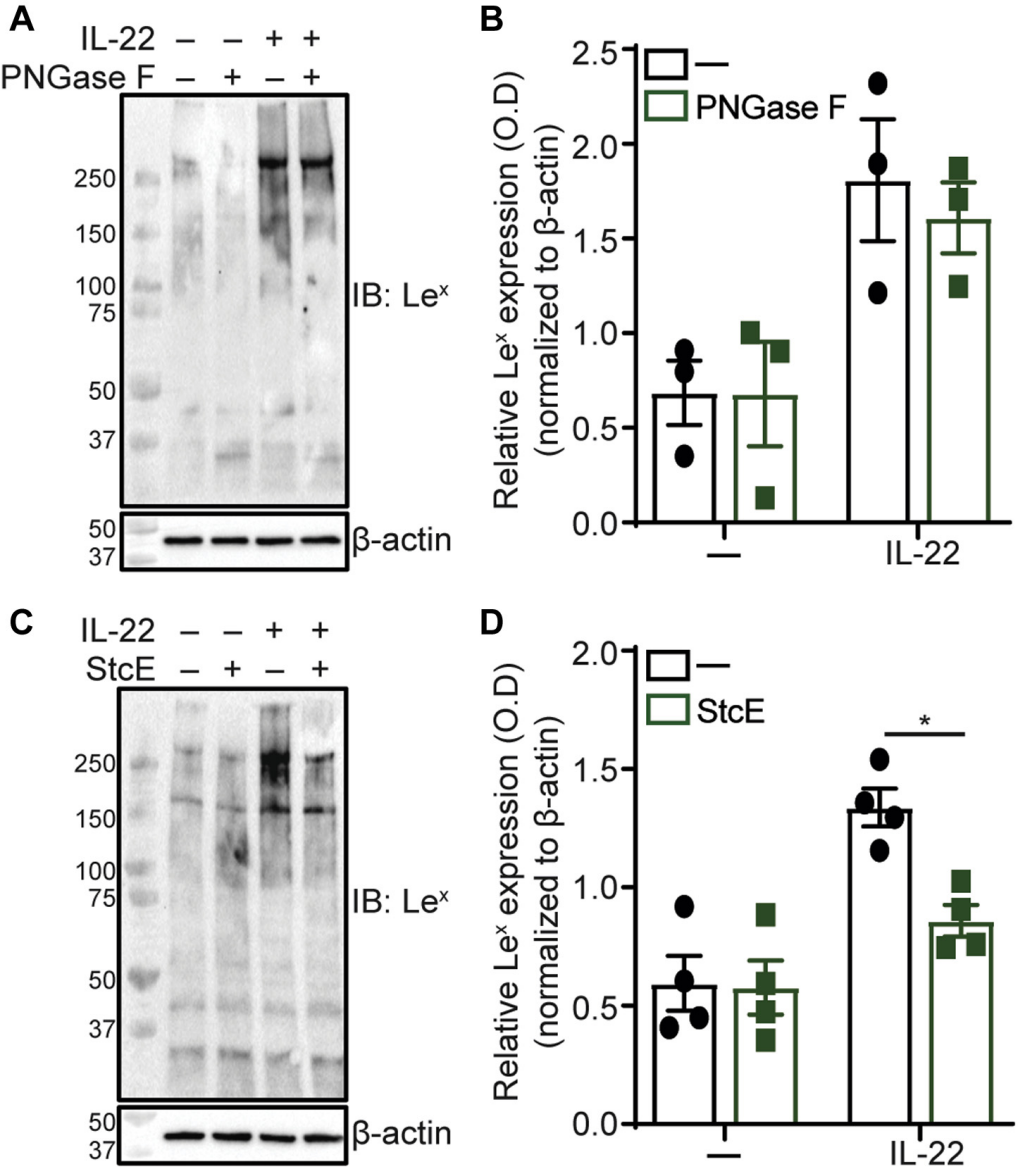
**IL-22 induces production of fucosylated *O*-linked glycans.***A*, differentiated Caco-2 BBe1 cells were incubated with 10 ng/ml rhIL-22 for 48 h before subsequent protein collection. *N*-linked glycans were released using PNGase F. Le^x^ antigen expression was assessed using Western blotting, and data are representative of three independent experiments. *B*, bar graphs show average quantified levels of Le^x^ antigen from replicate experiments and standard deviation. Statistical significance was assessed by one-way ANOVA with a Tukey post-hoc test. Data are not significant. *C*, differentiated Caco-2 BBe1 cells were incubated with 10 ng/ml of rhIL-22 for 48 h before the addition of 5 μg/ml StcE for 2 h. Proteins were collected and Le^x^ antigen expression was assessed as in (*A*). Data are representative of four independent experiments. *D*, bar graphs show average quantified levels of Le^x^ antigen from replicate experiments and standard deviation. Statistical significance was assessed as in (*B*). ∗*p* < 0.05.

Fucose in the α1-3-linkage can be displayed on either *N*- and *O*-linked glycans attached to proteins (28). To assess whether the IL-22-induced Le^x^ antigen was present on *N*- or *O*-linked glycans, differentiated Caco-2 BBe1 cells were first incubated with IL-22. Next, PNGase F (29) was used to release *N*-linked glycans in cell lysates, while StcE (30) was used on live cells to cleave peptides heavily modified with GalNAc-type *O*-glycans. We confirmed the effectiveness of the PNGase F treatment by monitoring the change in apparent molecular weight of LAMP1, a 40 kDa polypeptide that is normally modified with up to 18 *N*-linked glycans (Fig. S5). While PNGase F treatment reduced Le^x^ levels detected by immunoblot, this reduction was modest and not significant (Fig. 5, *A* and *B*). In contrast, StcE treatment led to a robust and statistically significant reduction in Le^x^ antigen levels (Fig. 5, *C* and *D*). Together, these results, combined with the observation that IL-22 induces expression of known *O*-glycosylated glycoproteins (*e.g.*, *DMBT1*, *MUC13*, *ICAM1*, etc.) (Table S1), implicate the involvement of IL-22 in the transcriptional regulation and downstream fucosylation of heavily *O*-glycosylated glycoproteins in human intestinal epithelial cells. Because PNGase F may not have equal accessibility to all *N*-linked glycans, our results do not exclude the possibility that IL-22 also induces α1-3 fucosylation of *N*-glycoproteins.

### B3GNT7 regulates epithelial fucosylation of *O*-linked glycans

**Figure 6:**
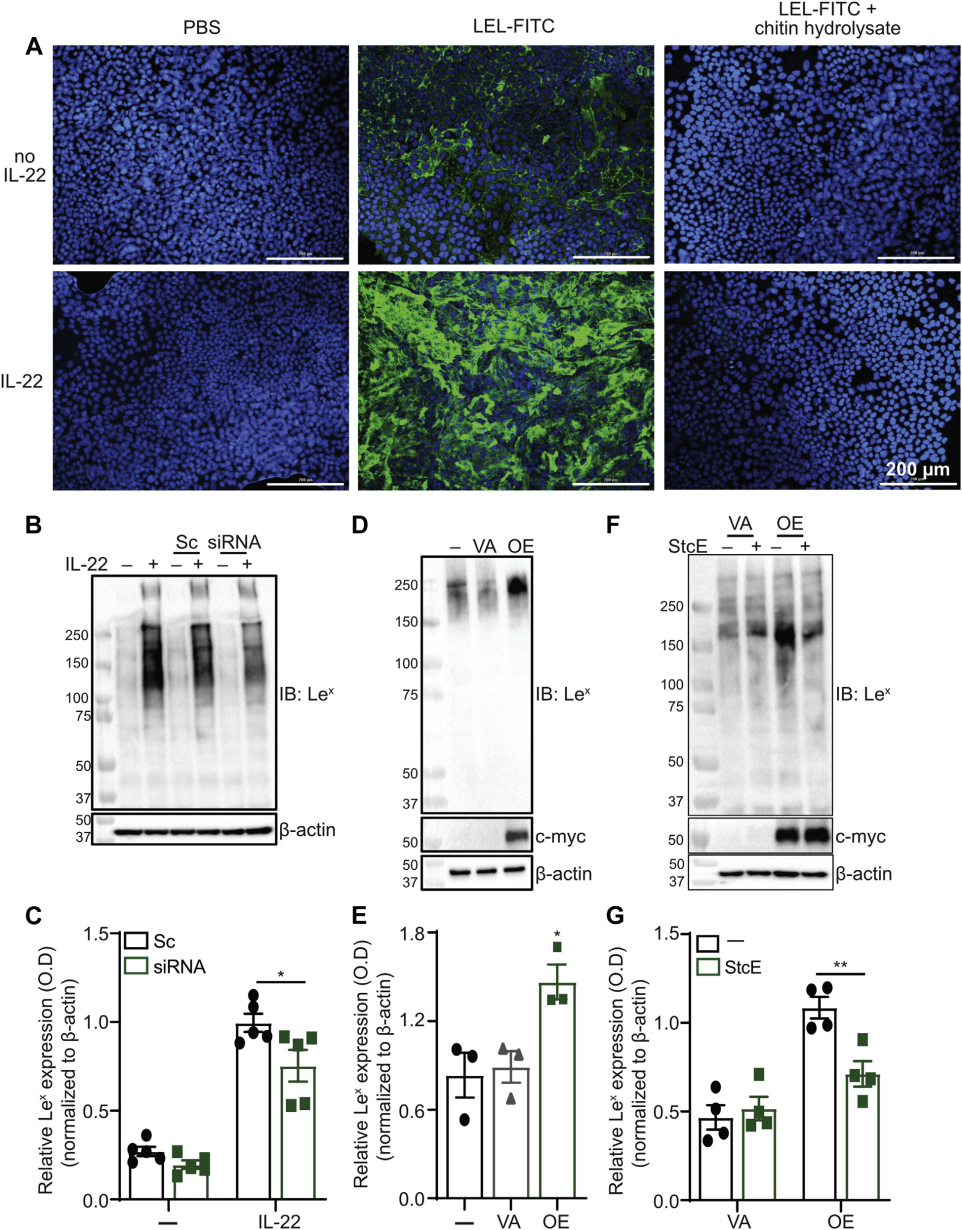
**B3GNT7 regulates epithelial fucosylation of *O*-linked glycans.***A*, differentiated Caco-2 BBe1 were incubated with rhIL-22 for 48 h, then stained with LEL-FITC in the presence or absence of chitin hydrolysate. Images presented are representative of three biological replicates. *B*, differentiated Caco-2 BBe1 cells were transfected with shRNA targeting *B3GNT7* (siRNA) or a scrambled control (Sc). Cells were incubated with rhIL-22 for 48 h and proteins were collected. Le^x^ antigen expression was assessed using Western blotting, and data are representative of five independent experiments. *C*, bar graph shows average quantified levels of Le^x^ antigen from replicate experiments. *D*, Caco-2 BBe1 cells were transfected with a plasmid encoding myc-tagged B3GNT7 (OE) or a vector alone control (VA). Proteins were collected and Le^x^ antigen expression was assessed as in (*A*). Data are representative of three independent experiments. *E*, bar graph shows average quantified levels of Le^x^ antigen from replicate experiments. *F*, Caco-2 BBe1 cells transfected with a plasmid encoding myc-tagged B3GNT7 (OE) or a vector alone control (VA) were incubated with 5 μg/ml StcE for 2 h. Proteins were collected and Le^x^ antigen expression was assessed as in (*A*). Data are representative of four independent experiments. *G*, bar graph shows average quantified levels of Le^x^ antigen from replicate experiments. For *C*, *E*, and *G*, error bars indicate standard deviation, and statistical significance was assessed by one-way ANOVA with a Tukey post-hoc test. ∗*p* < 0.05; ∗*p* < 0.01. B3GNT7, β1-3-*N*-acetylglucosaminyltransferase 7.

We next considered mechanisms by which IL-22 might induce increased α1-3-fucosylation. While the previously described IL-22-induced increase in α1-2-fucosylation in mice was attributed to increased *Fut2* expression (11), we did not detect IL-22-dependent changes in expression of FUTs capable of adding α1-3-fucose. Specifically, RT-qPCR analysis of *FUT4* and *FUT9,* genes encoding FUTs primarily responsible for Le^x^ synthesis (31), revealed no IL-22-dependent change in gene expression (Fig. S6). Additionally, inspection of RNA-sequencing data and subsequent validation using RT-qPCR revealed modest or no IL-22 dependent changes in expression of other FUT-encoding genes nor of genes encoding enzymes involved in GDP-fucose synthesis (Table S2). Thus, we considered other possible mechanisms to account for the increase in α1-3-fucosylation. Specifically, we hypothesized that the observed increase in α1-3-fucosylation could be due to increased production of glycan structures that are substrates for α1-3-fucosylation. *B3GNT7*, the glycosyltransferase gene that was most significantly upregulated by IL-22 treatment, encodes an *N*-acetylglucosaminyltransferase involved in the biosynthesis of polyLacNAc repeats of keratan sulfate (32, 33), which could potentially be fucosylated to produce the Le^x^ antigen. Indeed, we observed that Caco-2 BBe1 cells treated with IL-22 showed increased staining with *Lycopersicon esculentum* lectin (LEL), a lectin that recognizes GlcNAc residues in both chitin and polyLacNAc chains (Fig. 6*A*) (34). Inclusion of hydrolyzed chitin eliminated LEL staining, demonstrating the specificity of binding. We also considered whether additional genes might contribute to polyLacNAc biosynthesis but IL-22 treatment did not significantly affect expression of *B3GNT8,* another B3GNT family member expressed in this cell type (Fig. S7*A*), nor of *B4GALT1*, the B4GALT family member most abundantly expressed in these cells (Fig. S7*B*).

Finally, we performed experiments to explore the link between the IL-22-mediated increase in *B3GNT7* expression and IL-22-dependent induction of intestinal Le^x^ synthesis. First, we used siRNA to reduce expression of *B3GNT7* in Caco-2 BBe1 cells. Although the reduction in *B3GNT7* expression was modest (approximately 20%) (Fig. S8), we observed a commensurate and statistically significant decrease in IL-22-dependent Le^x^ expression (Fig. 6, *B* and *C*). Next, to assess whether epithelial fucosylation is directly regulated by B3GNT7, we analyzed the effect of ectopically overexpressing B3GNT7 in Caco-2 BBe1 cells and observed a statistically significant increase in Le^x^ expression (Fig. 6, *D* and *E*). Finally, we analyzed whether the B3GNT7-dependent increase in fucosylation occurred on *O*-linked glycans. Lysates from Caco-2 BBe1 cells overexpressing B3GNT7, like those from cells treated with IL-22, showed an StcE-dependent decrease in Le^x^ reactivity (Fig. 6, *F* and *G*). Together, these data implicate B3GNT7 in the regulation of production of fucosylated *O*-linked glycans and point to a mechanism in which the induced production of fucosylated epitopes is controlled by the availability of appropriate scaffold glycans.

## Discussion

In mice, IL-22 signaling has emerged as a key regulator of host glycosyltransferases, leading to increases in *Fut2*, *Mgat4a*, and *Mgat4b* expression, and the subsequent induction of intestinal epithelial fucosylation, which regulates interactions with resident microbes (10, 12, 35). However, whether IL-22 signaling regulates intestinal epithelial fucosylation in humans remained unexplored. In agreement with published data, we observed IL-22-dependent regulation of *FUT2* expression (Fig. 1*A*) and fucosylation (Figs. 4, S2 and S3) in human intestinal epithelial cells. However, unlike observations in the mouse, where IL-22 signaling regulates the synthesis of glycan structures decorated with α1-2- (11) or α1-3-linked fucose (Fig. S3*C*), IL-22 signaling in human intestinal epithelial cells only led to the enhanced synthesis of α1-3-fucosylated glycan structures (Fig. S3*A*) including the terminally α1-3-fucosylated carbohydrate epitope, Le^x^ (Fig. 4).

Le^x^ belongs to the human histo-blood group antigen system, and its synthesis is primarily attributed to the expression of *FUT4* and *FUT9*, encoding enzymes that catalyze the transfer of fucose to the acceptor substrate, GlcNAc in an α1-3-linkage (31, 36). Although we detected expression of these two genes in our experimental models, we failed to detect an IL-22-dependent regulation of *FUT4* and *FUT9* in both human and mouse intestinal epithelium (Fig. S5). In contrast, we detected an IL-22-dependent regulation of *B3GNT7* (Fig. 1). Importantly, IL-22-dependent regulation of *B3GNT7* was consistently observed in two human intestinal cell lines, in human-derived enteroid lines, and also in mouse ileal tissue. We note that IL-22-dependent induction of *B3GNT7* was moderate in Caco-2 BBe1 cells, which are commonly used to model of the small intestine, and more robust in T84 cells, which are morphologically and biochemically more similar to the colon (18); the mechanism underlying this difference may be worthy of further investigation. We did not observe IL-22-dependent regulation of *B3GNT7* in the mouse colon, a result that may be consistent with reports that parts of the large intestine are constitutively fucosylated in rodents (7, 28). However, IL-22-dependent regulation of *B3GNT7* has been shown in mouse colonic organoids (12).

*B3GNT7* encodes a β1-3-N-acetylglucosaminyltransferase (B3GNT) that catalyzes the transfer of GlcNAc from the uridine diphosphate (UDP)—GlcNAc donor to acceptor substrates in a β1-3-linkage (32, 37). Purified B3GNT7 has been shown to act on glycans with Gal at the nonreducing end. Among the acceptor glycans examined, B3GNT7 exhibited the highest observed activity toward a glycan comprising two LacNAc repeats with 4-*O*-sulfation on the Gal residues (32). Prior reports have demonstrated that B3GNT7 is involved in the synthesis of keratan sulfate glycosaminoglycans, which is attributed to its ability to extend sulfated and nonsulfated polyLacNAc chains commonly found in keratan sulfate chains (32, 33, 38). To extend polyLacNAc chains, B3GNT7 must act in concert with one or more galactosyltransferases to generate the repeating copolymer of Gal and GlcNAc. Notably, GlcNAc residues within polyLacNAc chains can serve as acceptors for α1-3-fucosylation, while terminal Gal residues are acceptors for α1-2-fucosylation (25). Since the synthesis of carbohydrate structures carrying the Le^x^ epitope requires a series of glycosyltransferases, we propose that IL-22 regulates the expression and activity of B3GNT7 to increase the availability of the acceptor substrate, GlcNAc, that can then be further modified by an α1-3-fucosyltransferase, such as FUT4 or FUT9. Although this possibility is in part supported by the ability of B3GNT7 to regulate Le^x^ synthesis in the absence of IL-22 (Fig. 6), further investigations are needed to fully elucidate the biochemical mechanisms involved. Because knockdown of B3GNT7 did not completely suppress IL-22-induced fucosylation, it remains possible that additional genes contribute as well. Regulation of fucosylation by controlling production of the polyLacNAc acceptor provides a mechanistic explanation for the difference in IL-22-induced fucose linkage observed in mouse *versus* human. Indeed, the roles of FUTs in biosynthesis of Lewis antigens are not strictly conserved between mouse and human (39). Therefore, we speculate that the relative abundance and redundancy of α1-3-fucosyltransferase expression may determine the amount of α1-3-fucosylated glycans produced in response to IL-22.

Our results demonstrate that much of the Le^x^ epitope produced in response to IL-22 is found on heavily *O*-glycosylated intestinal glycoproteins but does not exclude the possibility that some Le^x^ is attached to *N*-linked glycans (Fig. 5). In the intestine, the predominant heavily *O*-glycosylated glycoproteins are mucins (40). In mucosal glycans, fucose can be linked to a Gal by α1-2-linkage (generation of ABO blood group antigens) or to GlcNAc by α1-3-linkage (generation of Lewis antigens) (8), generating a plethora of fucose-decorated glycan structures. Interestingly, fucosylated epitopes can regulate commensal bacterial colonization by providing a nutritional advantage for distinct mucosa-associated bacteria (41, 42). For example, *Bacteroides thetaiotaomicron* and *Bifidobacterium bifidum*, common members of the intestinal microbiota, produce α-fucosidases that can liberate fucose from Lewis antigens that decorate host glycans present on mucosal glycans and utilize this fucose as a carbon source to support bacterial growth and colonization in the intestine (43, 44, 45). However, despite a growing body of evidence that demonstrates the importance of fucose present on mucins in intestinal homeostasis (41, 42), our understanding of the molecular mechanisms that govern mucin fucosylation is incomplete and is the basis of ongoing studies in our lab.

**Figure 7:**
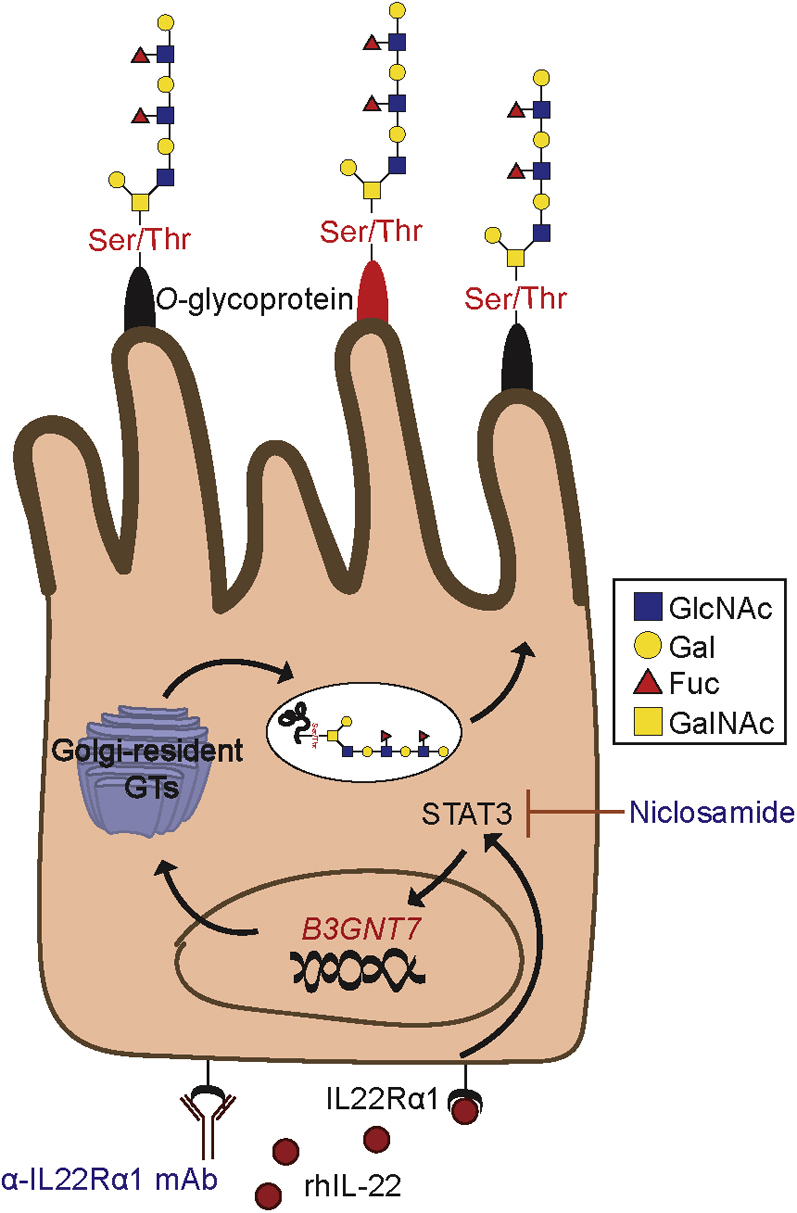
**Proposed mechanism for IL-22-induced increase in production of fucosylated *O*-linked glycans.** IL-22 signaling induces *B3GNT7* gene expression through activation of STAT3. The activity of B3GNT7 leads to increased production of polyLacNAc chains on *O*-linked glycans that provide substrates for fucosylation. B3GNT7, β1-3-*N*-acetylglucosaminyltransferase 7.

Together, the results presented here identify B3GNT7 as one intermediary in the IL-22 induction of intestinal fucosylation of *O*-linked glycans. The canonical IL-22-IL22Rα1-STAT3 pathway promotes transcription of *B3GNT7* and the subsequent synthesis of Le^x^-decorated glycan structures primarily present on heavily *O*-glycosylated intestinal epithelial glycoproteins (Fig. 7). More significantly, our discoveries uncovered a novel role for B3GNT7 in intestinal glycosylation. Prior reports have demonstrated the participation of B3GNT7 in the synthesis of keratan sulfate glycosaminoglycans in murine corneal tissue (38). However, despite its abundant expression in both mouse (37) and human (46) intestinal epithelium, a role for this enzyme in the regulation of intestinal glycosylation had not been identified. Notably, downregulation of *B3GNT7* gene expression has been linked to enhanced metastatic potential of colonic cancer cells (46). Furthermore, downregulation of *b3gnt7* gene expression was observed during dextran sodium sulfate (DSS)-induced colitis in mice (47). Therefore, understanding the intestinal cues that regulate B3GNT7 gene expression and function could provide new ideas about whether modulation of B3GNT7 can be used to alter intestinal glycosylation, effectively “healing” the intestinal glycome, to reduce microbial dysbiosis and resolve chronic inflammation in the gut.

## Experimental procedures

### Chemicals

Dimethyl sulfoxide (DMSO) was purchased from Sigma. 2-Fluoro-peracetyl- fucose [2F-Fuc; 98.8% pure] was purchased from EMD Millipore (Darmstadt); stock concentrations were made at 200 mM in DMSO. Niclosamide [niclo] was purchased from Selleckchem; stock concentrations were made at 5 mM in DMSO. Bovine serum albumin [BSA] was purchased from and Sigma. Carbohydrate-free blocking solution was purchased from Vector Laboratories. Complete ultra protease inhibitor tablets were purchased from Roche.

### Cell culture

The following reagents were used for general cell culture: heat-inactivated fetal bovine serum (HI-FBS), penicillin-streptomycin, TrypLE Express enzyme with phenol red (Gibco), Dulbecco’s Phosphate Buffered Saline (DPBS) (Sigma-Aldrich). Caco-2 BBe1 cells (ATCC) were maintained in Dulbecco’s Modified Eagle’s Medium (DMEM) (ATCC) supplemented with 10% HI-FBS (v/v), 1% penicillin-streptomycin and 10 μg/ml human transferrin (Sigma). T84 cells (ATCC) were maintained in 10% DMEM/F-12 medium (Gibco) supplemented with 5% (v/v) HI-FBS and penicillin-streptomycin. Both cell lines were maintained at 37 °C, 5% carbon dioxide in a water-saturated environment and were not used past passage number 32. The Countess automated cell counter (Life Technologies) was used for cell counting.

### Mice

*Stat3*^*fl/fl*^ and *Stat3*^*Δ*IEC^ (23) mice were bred and maintained as littermates at the University of Texas Southwestern Medical Center. About 9- to 20-week-old male and female mice were used for all experiments. The numbers of mice per group were chosen as the minimum needed to obtain biologically significant results. All experiments were performed using protocols approved by the Institutional Animal Care and Use Committees of the UT Southwestern Medical Center.

### Enteroid isolation from human tissue and culture

Human jejunum tissue was donated after informed consent by patients undergoing gastric bypass surgery. The specimens were obtained without any information about the patients and experiments on these tissues carried according to approval from the Ethical Review Board, Gothenburg, Sweden (ethics no. 583-17). The deidentified jejunum tissue was initially stripped of muscle and connective tissue using scissors to isolate the mucosa, before being stored in ice-cold PBS. Small biopsies were then taken and treated with PBS + antibiotic-antimycotic solution (1:100) (Thermo Fisher Scientific) for 4 × 2 min and then PBS + DTT (10 mM) for 3 × 2 min, before being incubated in PBS + EDTA (2 mM), 4 °C, on rotation for 1 h. The EDTA buffer was then removed and the tissue fragments were violently shaken in ice-cold PBS to isolate the stem cell crypts. The crypts were then embedded in Matrigel (hESC- Qualified Matrix) and plated in a 24-well tissue culture plate, before being cultured in Human Intesticult Organoid Growth Media (STEMCELL Technologies) supplemented with gentamicin. Following three passages, the spheroid cultures were considered to be stable enteroid lines and were used for experiments. In total, 10 μM Y27632 (STEMCELL) was also included in the media for the first 3 to 4 days after passage. Growth media was replaced every 2 to 4 days. Cultures were passaged every 1 to 2 weeks and incubated at 37 °C, 5% CO_2_. The three stable enteroid lines used for the qRT-PCR experiments were initially embedded in Matrigel in a 24-well tissue culture plate and cultured Human Intesticult Organoid Growth Media (components A plus B) supplement with gentamicin for 5 to 7 days. To differentiated the enteroids, component B of the Human Intesticult Organoid growth media was replaced with Advanced Dulbecco’s modified Eagle’s medium/F12 and Glutamax supplemented with 15 mM HEPES and incubated for a further 5 days before the addition of 10 ng/ml rhIL-22 (R&D Systems) for 4 h. In additional experiments, 5 μg/ml IL22Rα1 blocking antibody (Cat no. AF2770) or isotype control (R&D Systems) was also added prior to the addition of rhIL-22.

### Flow cytometry analysis

Enteroids were treated with IL-22 for 4 h and then everted as previously described (48). Everted enteroids were then subsequently incubated in TrypLE express for 30 min at 37 °C. Single cell suspensions were then resuspended in cold PBS and seeded into separate wells. All subsequent stain incubations occurred on ice. Samples were incubated for 10 min with the cell viability marker Zombie Red (BioLegend). Where applicable, cells were further treated with the biotinylated lectins: AAL or UEA I (Vector Laboratories) for 40 min, followed by incubation with streptavidin-BV421 (BioLegend) and anti-EpCAM-FITC (BioLegend) for 20 min. Other samples were also stained for anti-CD15-BV786 (BD Horizon) for 20 min. Flow cytometry buffer was 2 mM EDTA, 2% FBS in PBS. Cells were analyzed using a LSR Fortessa X-20 (BD Biosciences), and the data were analyzed using the software FlowJo V. 10.7 (FlowJo LLC). Events were gated to include only single cells, live cells, and EpCAM-positive cells.

### Cytokine treatment

Cells used for experiments were seeded at a density of 100,000 cells/well onto 12-mm-diameter Transwell permeable inserts with 0.4 μm pores (Corning). Caco-2 BBe1 cells were grown in supplemented DMEM for 14 days to achieve full differentiation prior to use, while T84 cells were grown in supplemented DMEM/F-12 for 10 days to achieve polarization prior to use. Cells were then placed in serum-free media (supplemented media without HI-FBS for each cell line) for 16 to 18 h before the addition of 10 ng/ml rhIL-22 to the basolateral compartment. In additional experiments, 5 μg/ml IL22Rα1 blocking antibody or isotype control (R&D Systems) was added to the basolateral compartment, while 0.5 μM niclosamide, 200 μM 2F-Fuc, or vehicle control was added to the apical compartment prior to the addition of rhIL-22.

### Cytokine time course

Caco2 BBe1 cells were seeded at a density of 100,000 cells/well onto 12-mm-diameter Transwell permeable inserts with 0.4 mm pores (Corning). Cells were grown in supplemented DMEM for 14 days to achieve full differentiation prior to use and were then placed in serum-free DMEM (supplemented without HI-FBS) for 16 to 18 h before the addition of 10 ng/ml rhIL-22 to the basolateral compartment. Treated cells were harvested at 2 h, 4 h, 6 h, and 8 h respectively, and qRT-PCR was performed to assess B3GNT7 mRNA levels at each time point.

### Cytokine dose response

Caco2 BBe1 cells were seeded at a density of 100,000 cells/well onto 12-mm-diameter Transwell permeable inserts with 0.4 mm pores (Corning). Cells were grown in supplemented DMEM for 14 days to achieve full differentiation prior to use and were then placed in serum-free DMEM (supplemented without HI-FBS) for 16 to 18 h before the addition of 0, 1, 10, and 100 ng/ml rhIL-22 to the basolateral compartment. Cells were harvested 4 h after cytokine treatment, and qRT-PCR was performed to assess B3GNT7 mRNA levels at each rhIL-22 dose.

### RNA sequencing

Total RNA from differentiated Caco-2 BBe1 cells was prepared using RNeasy Mini Kit (Qiagen), and contaminating DNA was removed using DNA-free DNA removal kit (Thermo Fisher Scientific). Samples were submitted to the Next-Generation Sequencing Core at UT Southwestern, where mRNA libraries were prepared using the Illumina TruSeq protocol and sequenced using Illumina NextSeq 500 with read configuration as 76 bp, single end. Each sample got approximately 42 million reads. Fastq files were checked for quality using fastqc (v0.11.2) (https://www.bioinformatics.babraham.ac.uk/projects/fastqc/) and fastq_screen (v0.4.4) (https://www.bioinformatics.babraham.ac.uk/projects/fastq_screen/). Fastq files were mapped to hg19 (UCSC version from igenomes) using STAR(v2.5.3a) (49). Read counts were generated using featureCounts (50), and the differential expression analysis was performed using edgeR (51). Genes with reads of log_2_ counts per million (CPM) >0 and *p* values <0.05 were considered differentially expressed. Volcano plot was made using EnhancedVolcano (https://www.bioconductor.org/packages/release/bioc/vignettes/EnhancedVolcano/inst/doc/EnhancedVolcano.html).

### Quantitative real-time PCR (qRT-PCR)

Total RNA from various intestinal epithelial cell lines, human enteroids, and mouse intestinal tissue was prepared using RNeasy Mini Kit (Qiagen). RNA was reverse transcribed using qScript cDNA Synthesis Kit (Quantabio), and cDNA was used for qRT-PCR analysis using SsoAdvanced Universal SYBR Green Supermix (Bio-Rad Laboratories) on a CFX384 Touch Real-Time PCR Detection System (Bio-Rad Laboratories). The following primer sets were used for amplification: Human *B3GNT7*-F, 5′-CCACTAACTGCTCAGCCAAT; *B3GNT7*-R, 5′-GATGACCGACTTGACAACCA; human *GAPDH*-F, 5′-GCAAATTCCATGGCACCGT; *GAPDH*-R, 5′-TCGCCCCACTTGATTTTGG; mouse *B3gnt7*-F, 5′-CCTACGAGGACCGTCTCTAT; *B3gnt7*-R, 5′-TGGGACAGTAAATGTCGAGC; mouse *Fut2*-F, 5′-CCCACTTCCTCATCTTTGTCTTT; *Fut2*-R, 5′- TTTGAACCGCCTGTAATTCCTT; mouse *Gapdh*-F, 5′-TGTCCGTCGTGGATCTGAC; *Gapdh*-R, 5′-CCTGCTTCACCACCTTCTTG.

### Nucleofection

Amexa Cell Line Nucleofector Kit T (Lonza) was used for knock down and overexpression experiments per manufactures’ recommendations. Briefly, 1 × 10^6^ cells were resuspended in 100 μl of transfection buffer containing 5 μg *B3GNT7* (NM_145236) human tagged ORF clone [Cat no. RC218503] or pCMV6-entry tagged clone vector [Cat no. PS100001] (OriGene), and 30 nM *B3GNT7* stealth siRNA [Cat no. HSS132466] or Stealth RNAi Negative Control Duplex Medium GC Duplex [Cat no. 12935-300] (Thermo Fisher Scientific). The cell/plasmid mixtures, in 1 cm cuvettes, were nucleoporated using the Nucleofector 2b Device (Lonza) according to predefined program B-024. Following nucleofection, cells for overexpression experiments were seeded into a 6-well tissue culture plate for 48 h before analysis, while cells for knockdown experiments were seeded in 24-mm-diameter Transwell permeable inserts with 0.4 μm pores (Corning) and allowed to differentiate for 6 days before cytokine treatment.

### Western blotting

Total protein from differentiated Caco-2 BBe1 cells was prepared using RIPA buffer (50 mM Tris-HCl, pH = 8; 150 mM NaCl; 0.01% (v/v) SDS; 0.5% (v/v) sodium deoxycholate; 1% (v/v) IGEPAL CA-630; 1× protease inhibitor). Protein content was quantified with a BCA assay kit (Thermo Fisher Scientific) against a BSA standard curve for normalization. For protein resolution, 10 to 15 μg of lysate was denatured in 1× SDS loading dye (250 mM Tris-HCl, pH 6.8; 0.08% (w/v) SDS; 0.0004% (w/v) bromophenol blue; 40% (v/v) glycerol; 5% (v/v) 2-mercaptoethanol) for 5 min at 95 °C. The samples were separated by SDS-PAGE, and protein was transferred to a PVDF membrane (ED Millipore) using the Trans-Blot SD Semi-Dry Transfer Cell (Bio-Rad Laboratories), according to the manufacturer’s guidelines. After blocking at room temperature for 1 h, membranes were probed overnight at 4 °C with 1:1000 dilution of mouse anti-human CD15 clone HI98 [anti-Le^x^] (Cat no. 555400BD); mouse anti-human Lamp1 (Cat no. 611042); (both from BD Biosciences), anti-Myc Tag (Cat no. 2276S); (Cell Signaling Technologies), biotinylated UEA-I or biotinylated LTL lectin (both from Vector Laboratories). When probing with an antibody, membranes were blocked and incubated in 5% BSA prepared in 1× TBS containing 0.05% (v/v) Tween-20 (TBS-T). When probing with a lectin, membranes were blocked and incubated with 1× Carbohydrate-free solution prepared in 1× TBS-T. Specific binding of antibodies and lectins was detected with HRP-conjugated secondary antibodies or streptavidin (1:5000 dilution) using the SuperSignal West Pico PLUS Chemiluminescent substrate (Thermo Fisher Scientific) and ChemiDoc MP Imaging system (Bio-Rad Laboratories). Membranes were stripped in Restore Western Blot Stripping Buffer (Thermo Fisher Scientific) for 15 min at room temperature before reprobing with HRP-conjugated anti-β-actin (Cat no. 5125S; Sigma, 1:1000 dilution) overnight at 4 °C.

### *N*-glycan release

*N*-linked glycans were released using PNGase F (Cat no. P0704L; NEB) per manufactures’ recommendations. Briefly, protein lysates from IL-22-treated Caco-2 BBe1 cells were incubated with 500 units of PNGase F for 16 h at 37 °C, and samples were further analyzed using Western blotting.

### O-glycosylated peptide cleavage

Densely O-glycosylated peptides were cleaved using the protease, StcE (kindly provided by Dr Carolyn Bertozzi). Following cytokine treatment or nucleofection to overexpress B3GNT7, cells were washed with prewarmed DPBS one time and then incubated with 5 μg/ml StcE in serum-free media for 2 h at 37 °C. Protein was collected, and samples were further analyzed using Western blotting.

### Immunofluorescence (mouse tissue)

Segments of unflushed ileum from each mouse were prepared as previously described (52). To prepare samples for staining, slides were dewaxed and cleaned in the following solutions for indicated times: one time in xylene for 5 min, one time in xylene for 3 min, two times in 100% ethanol for 3 min, two times in 95% ethanol for 3 min, two times in 70% ethanol for 3 min and two times in water for 5 min. Slides were blocked in 1× Carbohydrate-free solution prepared in 1× DPBS containing 0.05% (v/v) Tween-20 (DPBS-T) at room temperature for 1 h and then washed three times in DPBS-T. After drying, 10 μg/ml FITC-conjugated UEA I lectin [Cat no. FL-1061] and 20 μg/ml FITC-conjugated LTL lectin [FL-1321] (Vector Laboratories) diluted in 1× Carbohydrate-free solution were added to the slides and incubated overnight at 4 °C, in the dark. For specificity controls, FITC-conjugated UEA 1 and LTL lectins were incubated for 1 h at room temperature in the presence of 100 mM l-fucose [Cat no. MF06710] (Biosynth Carbosynth), and the aforementioned staining procedure was performed. Following overnight incubation, slides were washed three times in DPBS-T, dried, and mounted with DAPI Fluormount-G (Southern Biotech) and sealed. Samples were left to set for at least 24 h at room temperature before imaging on Cytation 5 Cell Imaging Multi-Mode Reader (BioTek). Images were generated using 10× magnification.

### Immunofluorescence (human cells)

Caco2 BBe1 cells were seeded at a density of 300,000 cells/well onto 24 mm-diameter transwell permeable inserts with 0.4 mm pores (Corning). Cells were grown in supplemented DMEM for 14 days to achieve full differentiation prior to use. Cells were then placed in serum-free media for 16 to 18 h before the addition of 10 ng/ml rhIL-22 to the basolateral compartment. After 48 h, cells were washed twice with DPBS and fixed with 4% PFA [Cat no. 28908] (Thermo Fisher Scientific) for 10 min at room temperature. Cells were then washed twice with DPBS and incubated with 5 mg/ml FITC-LEL [Cat no. FL-1171-1] (Vector Laboratories), diluted in DPBS, for 1 h at room temperature in the dark. For a specificity control, FITC-LEL was incubated with a 1:4 dilution of chitin hydrolysate [Cat no. SP-0090-10] (Vector Laboratories) for 1 h at room temperature in the dark, and the same staining procedure was performed. Again, cells were washed twice with DPBS. Cells were scraped off the transwell insert into 40 ml DPBS and transferred to a glass slide. Cells were mounted with 4 μl VECTASHIELD Antifade Mounting Medium with DAPI [LS-J1033] (LSBio), and the sample was covered with a glass cover slip. Cells were then imaged on the Cytation 5 Cell Imaging Multi-Mode Reader (BioTek). Images were generated using 10× magnification.

### Statistical analysis

Data are presented as mean ± standard deviation, unless otherwise indicated. Statistical significance was determined as indicated in each figure legend using GraphPad Prism 8.0 (GraphPad Software). For cell culture experiments, parametric tests (*t* test or one-way ANOVA) were applied; for analysis of mouse samples, a nonparametric test (Mann-Whitney) was used. Outliers were determined by using the Grubbs test. Values were considered significant at a *p* value of less than 0.05.

## Data availability

RNA-seq data were deposited to Gene Expression Omnibus (GSE154889).

## Supporting information

This article contains supporting information.

## Conflict of interest

The authors declare that they have no conflicts of interest with the contents of this article.
