# Supplementary material for: Interleukin-22 regulates B3GNT7 expression to induce fucosylation of glycoproteins in intestinal epithelial cells
Source: J Biol Chem. 2021 Dec 2;298(2):101463. doi: 10.1016/j.jbc.2021.101463 (PMC8808068; doi:10.1016/j.jbc.2021.101463)
Supplement: Supplemental Figures S1–S8 [file mmc1.pdf]

SUPPORTING INFORMATION

Experimental procedures

Quantitative real-time PCR (pRT-PCR) primers used for gene expression analysis (F – forward primer; R – reverse primer)

Gene	Primer (5’ → 3’)
B3GNT8 - F	CCTTTGAGGACGTCTACACTGG
B3GNT8 - R	AGGTTGCGGAAAGCACAGTGGT
B4GALT1 - F	GTATTTTGGAGGTGTCTCTGCTC
B4GALT1 - R	GGGCGAGATATAGACATGCCTC
FUT1 - F	TATGACCATTGGCACCTTCG
FUT1 - R	TCAAGGCTTAGCCAATGTCC
FUT2 - F	CTACCACCTGAACGACTGGATG
FUT2 - R	AGGGTGAACTCCTGGAGGATCT
FUT3 - F	GCCGACCGCAAGGTGTAC
FUT3- R	TGACTTAGGGTTGGACATGATATCC
FUT4 - F	GGGTTTGGATGAACTTCGAGTCG
FUT4- R	GGTAGCCATAAGGCACAAAGACG
FUT3/5/6 - F	CCGACTACATCACCGAGAAGCT
FUT3/5/6- R	GAACCTCTCGTAGTTGCTTCTGC
FUT7 - F	GAATGAGAGCCGATACCAACGC
FUT7- R	TAGCGGTCACAGATGGCACAGA
FUT8 - F	GACAGAACTGGTTCAGCGGAGA
FUT8- R	GCAGTAGACCACATGATGGAGC
FUT9 - F	TGGAATCAGCCAGCTCTGTGCT
FUT9 - R	CGTTGTGAGATGGCATCCTTGG
FUT10 - F	CTAACCAGCGACTTCTGACAGC
FUT10 – R	CCCATCTTTTGGGTGGTAAGCC
GMDS - F	GGAGAAAGGCTATGAGGTCC
GMDS - R	CTCAGCGAGGTCAAAGGAAA
TSTA3 - F	ATCTCACGGATACAGCACAG
TSTA3 - R	GAAGATACAGGTGGACAGGC
FPGT - F	GCAATAACAGCGGCTGATGAA
FPGT - R	AAGGGCACAAAGTGTTGATCCTC
Fut4 - F	CAAAGCCCTGGAGACCGTAGGT
Fut4 - R	CGCTCCTGGAATAGAGGAAGCC

Fut9 - F	CAAATCCCATGCGGTCCTGAT
Fut9 - R	TGCTCACCGTCAAGAAGCCATAA

Supporting figures and figure legends

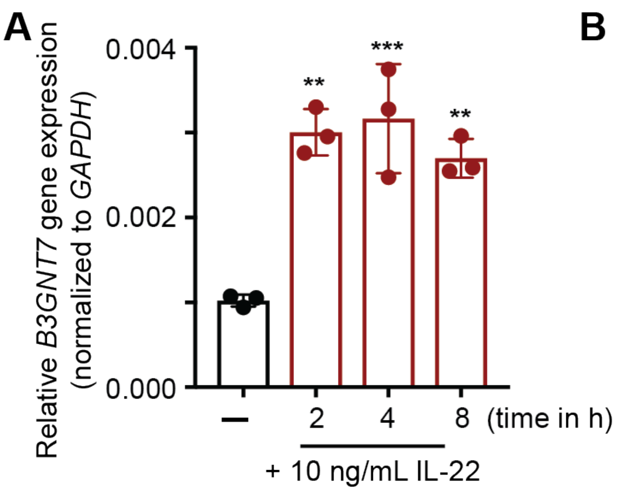

Figure S1: Time course and concentration dependence of IL-22-induced *B3GNT7* expression. (A) Differentiated Caco-2 BBe1 cells were incubated with 10 ng/mL rhIL-22 for the time indicated before subsequent RNA isolation. *B3GNT7* gene expression were assessed using qRT-PCR and data were normalized to *GAPDH* levels for each condition. (**B**) Differentiated Caco-2 BBe1 cells were incubated for 4 h with rhIL-22 at the concentrations indicated before subsequent RNA isolation. *B3GNT7* gene expression were assessed using qRT-PCR and data were normalized to *GAPDH* levels for each condition. For both panels, symbols represent individual replicates (n = 3) and error bars represent standard deviation. Statistical significance was assessed by unpaired, two-tailed *t*-test.

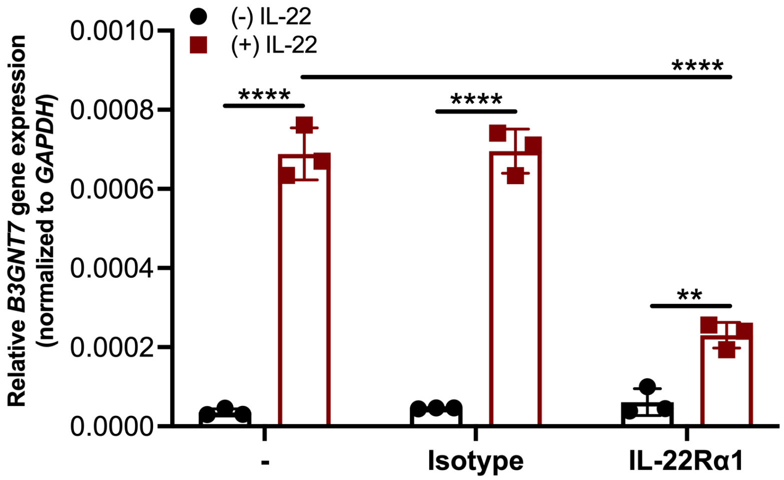

Figure S2: IL-22 signaling regulates *B3GNT7* gene expression in colonic epithelial cells. Polarized T84 cells were incubated with 5 μg/mL IL22Rα1 blocking antibody (IL22Rα1) or isotype control (Iso) for 1 h before the addition of rhIL-22 for 4 h before subsequent RNA isolation. *B3GNT7* gene expression was assessed using qRT-PCR and data were normalized to *GAPDH* levels for each condition. Symbols represent individual replicates (n = 3) and error bars show standard deviation. Statistical significance was assessed by one-way ANOVA with a Tukey post-hoc test. **** $p < 0.0001$.

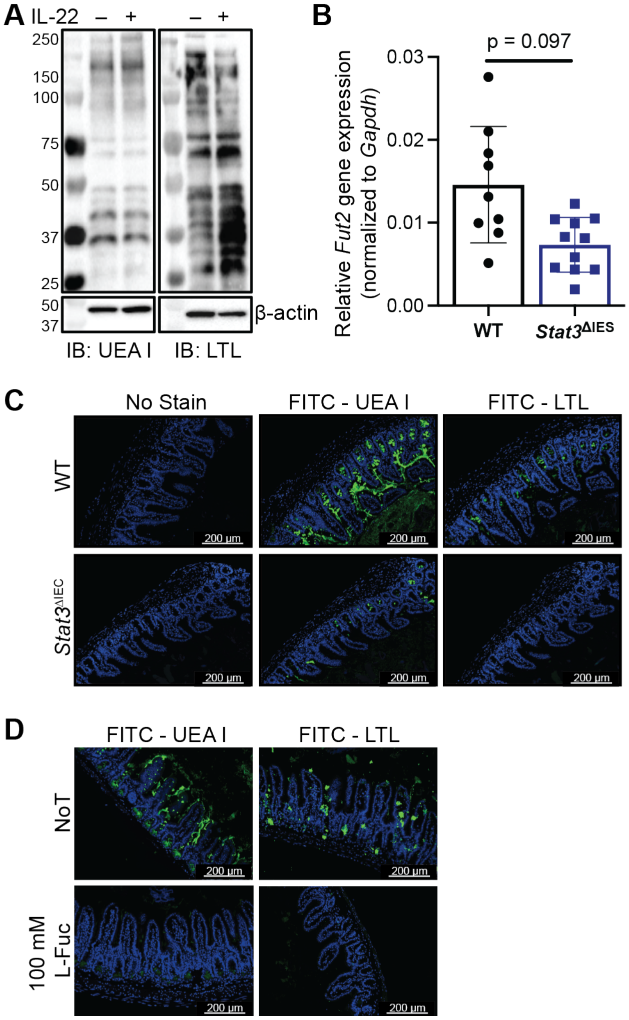

Figure S3: Regulation of intestinal epithelial *Fut2* gene expression and fucosylation. (A) Differentiated Caco-2 BBe1 cells were incubated with rhIL-22 for 48 h before subsequent protein collection. Changes in fucosylation were assessed using UEA I (detects α1-2-fucosylation) and LTL (detects α1-3-fucosylation) lectins. Data are representative of at least three independent experiments. **(B)** *Fut2* gene expression in ileum of WT and *Stat3*$^{\Delta IEC}$ mice was quantified by qRT-PCR and normalized to *Gapdh* levels for each sample. Data are from male and female mice, and symbols represent individual mice (n = 9 biologically independent animals for WT; n = 11 biologically independent animals for *Stat3*$^{\Delta IEC}$). Error bars show standard deviation and statistical significance was assessed using a two-tailed Mann-Whitney test. **(C)** Ileal tissue sections of WT or *Stat3*$^{\Delta IEC}$ were stained with UEA I and LTL lectins (n = 3 biologically independent animals per group). Scale bars, 200 μm. Data are representative of three independent experiments. **(D)** Ileal tissue sections of a WT mouse were stained with UEA I and LTL lectins in the presence or absence of 100 mM L-fucose. Scale bars, 200 μm. Data are representative of three independent experiments.

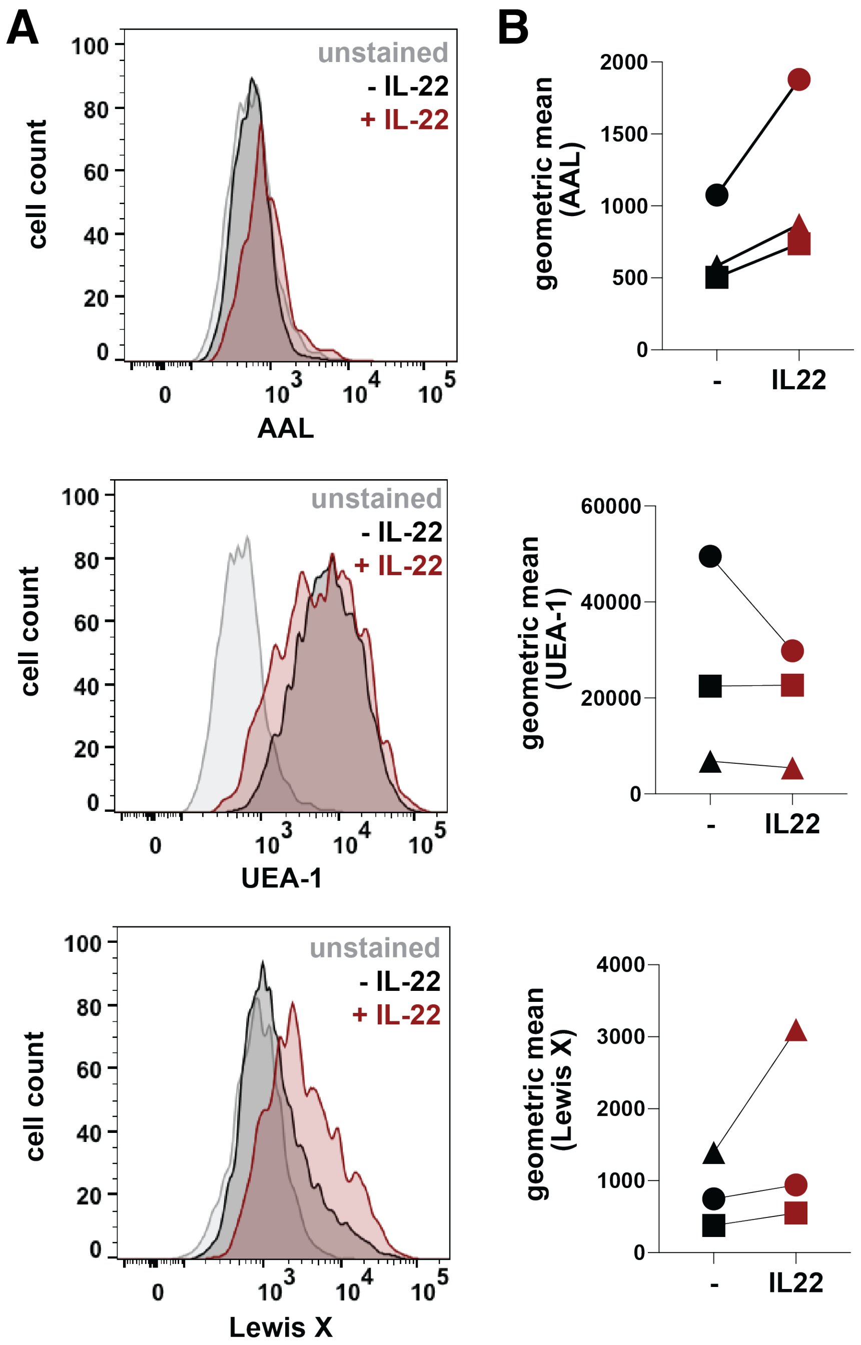

Figure S4: Effects of IL-22 signaling on cell surface glycosylation in human enteroids. Differentiated enteroid cultures derived from three different individuals were after eversion incubated with 10 ng/mL rhIL-22 for 4 h before being dissociated into single suspensions, and analyzed by flow cytometry. (**A**) Representative flow cytometry analysis of binding of the AAL lectin, UEA I lectin, and anti-Le^x antibody to the EpCAM-positive subset of enteroids derived from a single individual. (**B**) Combined data for flow cytometry analysis of the effect of rhIL-22 treatment on antibody and lectin binding to enteroids derived from three individuals (represented by squares, circles and triangles).

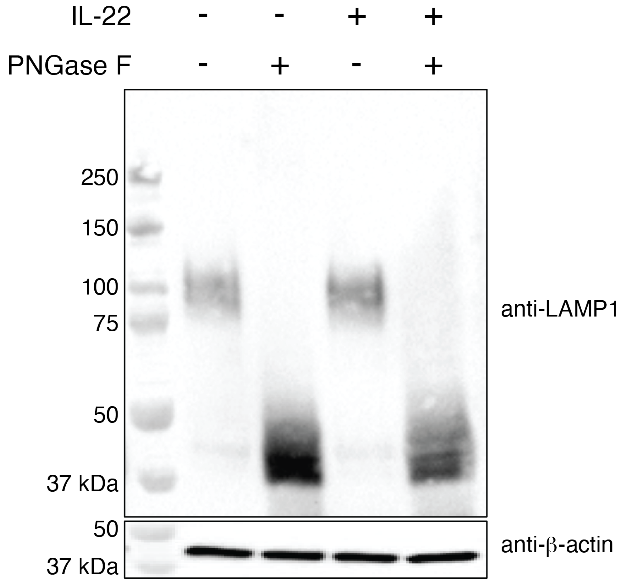

Figure S5: PNGase F treatment effectively cleaves N-linked glycans from LAMP1. Protein lysates from IL-22-treated Caco-2 BBe1 cells were incubated with 500 units of PNGase F for 16 h at 37 °C, and samples were further analyzed by immunoblot using an anti-LAMP1 antibody. The predicted molecular weight of the unmodified LAMP1 polypeptide is ~40 kDa. The blot presented is representative of three biological replicates.

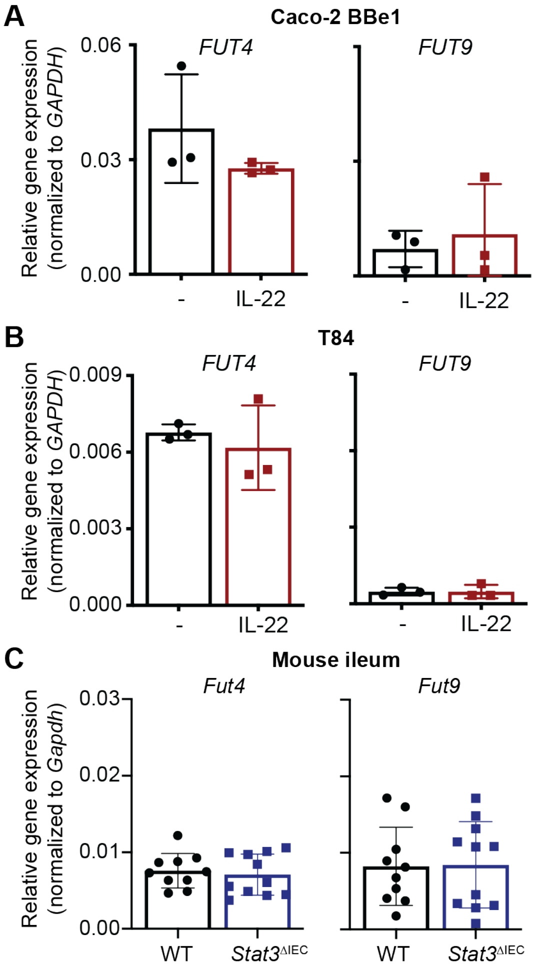

Figure S6: *FUT4* and *FUT9* gene expression in intestinal epithelium. (A) Differentiated Caco-2 BBe1 cells or **(B)** polarized T84 cells were incubated with 10 ng/mL rhIL-22 for 4 h and RNA was isolated. *FUT4 and FUT9* gene expression was assessed using qRT-PCR and data were normalized to *GAPDH* levels for each condition. Symbols represent individual replicates (n = 3) and error bars show standard deviation. Statistical significance was assessed by unpaired, two-tailed *t*-test. Differences were not statistically significant. **(C)** *Fut4 and Fut9* gene expression in ileum of WT and *Stat3*$^{\Delta IEC}$ mice was quantified by qRT-PCR and normalized to *Gapdh* levels for each sample. Data are from male and female mice, and symbols represent individual mice (n = 9 biologically independent animals for WT; n = 11 biologically independent animals for *Stat3*$^{\Delta IEC}$). Error bars show standard deviation and statistical significance was assessed using a two-tailed Mann-Whitney test. No statistically significant differences between WT and *Stat3*$^{\Delta IEC}$ samples were detected.

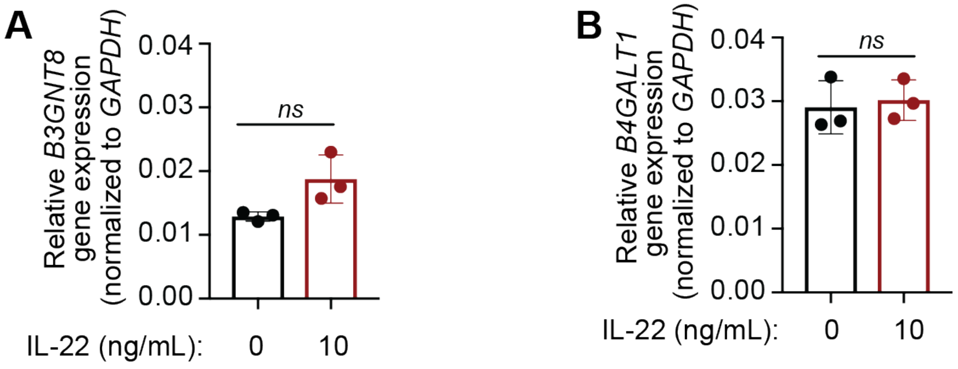

Figure S7: IL-22 does not regulate expression of B3GNT8 or B4GALT1. Differentiated Caco-2 BBe1 cells were incubated with 10 ng/mL rhIL-22 for 4 h before subsequent RNA isolation. *B3GNT8* (**A**) and *B4GALT1* (**B**) gene expression were assessed using qRT-PCR and data were normalized to *GAPDH* levels for each condition. For both panels, symbols represent individual replicates (n = 3) and error bars represent standard deviation. Statistical significance was assessed by unpaired, two-tailed *t*-test.

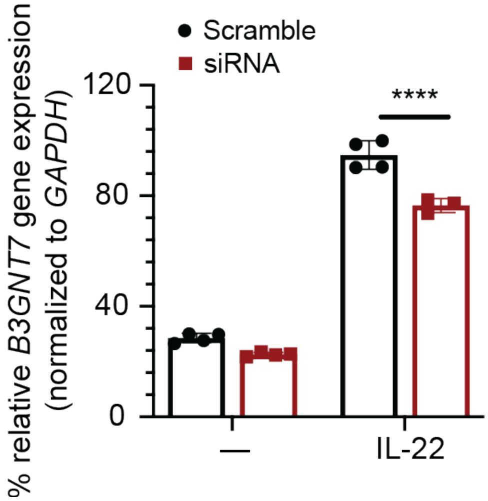

Figure S8: Reduced *B3GNT7* gene expression in differentiated Caco-2 BBe1 cells. Caco-2 BBe1 were transfected with shRNA targeting *B3GNT7* (siRNA) or a scrambled control (Sc). Following differentiation, cells were incubated with 10 ng/mL rhIL-22 for 4 h before subsequent RNA isolation. *B3GNT7* gene expression was assessed using qRT-PCR and data were normalized to *GAPDH* levels for each condition (n = 4) and statistical significance was assessed by one-way ANOVA with a Tukey post-hoc test. Error bars show standard deviation. **** $p < 0.0001$.
